# Draft genomes of *Lactiplantibacillus pentosus* PL0028 and PL0050 isolated from Philippine fermented foods

**DOI:** 10.1128/mra.00662-26

**Published:** 2026-08-24

**Authors:** Joshua T. Veluz, Paul Christian T. Gloria, Ma. Jhanessa A. Luague, Kyla C. Austria, Maria Auxilia T. Siringan, Mary Ann Cielo Relucio-San Diego

**Affiliations:** 1 Natural Sciences Research Institute, College of Science, University of the Philippines Diliman 551109, Quezon City, Philippines; Wellesley College, Wellesley, Massachusetts, USA

**Keywords:** burong bangus, burong hipon, genomics, lactic acid bacteria, *Lactiplantibacillus pentosus*, Philippine fermented foods

## Abstract

We report the draft genomes of *Lactiplantibacillus pentosus* PL0028 and PL0050, isolated from Philippine fermented rice–milkfish and rice–shrimp mixtures, respectively. This genome announcement contributes to the growing genomic literature on lactic acid bacteria from Philippine fermented foods.

## ANNOUNCEMENT

Philippine fermented foods harbor diverse lactic acid bacteria (LAB) (1), yet only a limited number of studies (2–5) have applied whole-genome sequencing to characterize these microbes. Genomics provides stronger taxonomic resolution and enables the identification of genetic features associated with survival and functionality (6) within fermented foods. Such information supports the scientific and cultural value of locally derived LAB from Philippine fermented foods. This genome announcement reports two *Lactiplantibacillus pentosus* isolates from distinct Philippine fermented foods, confirms their species identity, and expands genomic resources for LAB from local fermented foods, providing references for future safety and functional analyses.

The bacterial isolates were obtained from fermented rice–milkfish (*burong bangus*) and rice–shrimp (*burong hipon*) mixtures by spread-plating 100 µL of serially diluted samples, up to 10^−^⁵ in normal saline, on De Man, Rogosa, and Sharpe (MRS) agar (7). Isolation was performed at the Microbiological Research and Services Laboratory, Natural Sciences Research Institute, University of the Philippines Diliman (14°39′08.16″ N, 121°04′08.39″ E). Plates were incubated at 35°C for 48–72 h under microaerophilic conditions (8, 9). Colonies were subsequently selected and purified. The purified isolates are maintained as glycerol stocks at −83°C in the University of the Philippines Diliman Culture Collection. The isolates were characterized based on colony and cellular morphology and catalase and oxidase tests, confirming characteristics consistent with LAB.

For genomic DNA extraction, the isolates were cultivated in MRS medium at 35°C for 24 h under microaerophilic conditions (4). The DNA was extracted using the DNeasy PowerSoil Pro Kit (QIAGEN, Germany) following the manufacturer’s handbook (HB-2495), with extended vortexing of the PowerBead Pro tube. This kit was used based on the laboratory’s consistent DNA yield suitable for whole-genome sequencing. DNA extracts (>100 ng/µL) were submitted to Macrogen, Inc. (South Korea) for whole-genome sequencing. Libraries were prepared using the TruSeq Nano DNA Library Kit (Illumina, USA) and sequenced on the Illumina NovaSeq 6000 platform using 151 bp paired-end reads with an expected throughput of 2 Gb.

Unless otherwise specified, all bioinformatics tools were run using default parameters. The raw sequencing data for PL0028 consisted of 3,322,221,668 total bases, 22,001,468 total reads, and a GC content of 45.2%, whereas PL0050 yielded 3,284,859,134 total bases, 21,754,034 total reads, and a GC content of 45.4%. Raw reads were quality-trimmed using Trimmomatic version 0.36 (10) and subsequently assembled using SPAdes version 4.1.0 (11) through Unicycler version 0.5.1 (12) in bold bridging mode. The genome characteristics and closest taxonomic matches, assigned by NCBI using average nucleotide identity (ANI)-based genome comparison of the assemblies, are summarized in Table 1.

**TABLE 1:** Genome characteristics and closest taxonomic matches of PL0028 and PL0050

Parameters		PL0028	PL0050
CheckM version 1.2.4 (13)	Completeness (%)	99.35	99.35
	Contamination (%)	2.21	2.21
QUAST version 5.3.0 (14)	Genome size (bp)	3,649,899	3,649,594
	Number of contigs	115	108
	N50 (bp)	78,830	88,350
	L50	14	11
	GC (%)	46	46
Best ANI match		*Lactiplantibacillus pentosus* DSM 20314 (GCA_003641185.1), 99.83% ANI	*Lactiplantibacillus pentosus* DSM 20314 (GCA_003641185.1), 99.83% ANI

The draft genome assemblies showed >90% completeness based on CheckM analysis, with CheckM-estimated contamination of <5%. Both genomes exceeded the 95% ANI species-level cutoff (15), confirming the isolates as *L. pentosus*. Using NCBI’s Prokaryotic Genome Annotation Pipeline version 6.10 (16), we predicted PL0028 and PL0050 to contain 3,385 genes (3,229 protein coding) and 3,378 genes (3,227 protein coding), respectively.

## Data Availability

All sequence data, including the raw reads (SRX30104964 and SRX30104967) and genome assemblies (GCA_053748715.1 and GCA_053748595.1) of PL0028 and PL0050, were deposited under NCBI BioProject PRJNA1305679.
